# Discovery of Novel Markers for Identifying Cognitive Decline Using Neuron-Derived Exosomes

**DOI:** 10.3389/fnagi.2021.696944

**Published:** 2021-08-26

**Authors:** Jiacheng Zhong, Xiaohu Ren, Wei Liu, Shuqi Wang, Yuan Lv, Lulin Nie, Rongying Lin, Xiaoping Tian, Xifei Yang, Feiqi Zhu, Jianjun Liu

**Affiliations:** ^1^Shenzhen Key Laboratory of Modern Toxicology, Shenzhen Medical Key Discipline of Health Toxicology (2020–2024), Shenzhen Center for Disease Control and Prevention, Shenzhen, China; ^2^Key Laboratory of Molecular Epidemiology of Hunan Province, School of Medicine, Hunan Normal University, Changsha, China; ^3^Cognitive Impairment Ward of Neurology Department, The Third Affiliated Hospital of Shenzhen University Medical College, Shenzhen, China

**Keywords:** Alzheimer’s disease, mild cognitive impairment, proteomics, neuron-derived exosomal markers, bioinfomatics analysis

## Abstract

Alzheimer’s disease (AD), the predominant cause of late-life dementia, has a multifactorial etiology. Since there are few therapeutic options for symptomatic AD, research is increasingly focused on the identification of pre-symptomatic biomarkers. Recently, evaluation of neuron-derived exosomal markers has emerged as a promising novel approach for determining neuronal dysfunction. We aimed to identify novel neuron-derived exosomal markers that signify a transition from normal aging to Mild Cognitive Impairment (MCI) and then to clinically established AD, a sequence we refer to as AD progression. By using a Tandem Mass Tag-based quantitative proteomic approach, we identified a total of 360 neuron-derived exosomal proteins. Subsequent fuzzy c-means clustering revealed two clusters of proteins displaying trends of gradually increasing/decreasing expression over the period of AD progression (normal to MCI to AD), both of which were mainly involved in immune response-associated pathways, proteins within these clusters were defined as bridge proteins. Several differentially expressed proteins (DEPs) were identified in the progression of AD. The intersections of bridge proteins and DEPs were defined as key proteins, including C7 (Complement component 7), FERMT3 (Fermitin Family Member 3), CAP1 (Adenylyl cyclase-associated protein 1), ENO1 (Enolase 1), and ZYX (Zyxin), among which the expression patterns of C7 and ZYX were almost consistent with the proteomic results. Collectively, we propose that C7 and ZYX might be two novel neuron-derived exosomal protein markers, expression of which might be used to evaluate cognitive decline before a clinical diagnosis of AD is warranted.

## Introduction

Dementia manifests *via* progressive cognitive impairment leading to patient dependency or even death (Duong et al., 2017). According to an analysis of dementia prevalence in 2015, there were some 47 million dementia patients around the world, and this number was predicted to reach 131 million by the mid-21st century (Arvanitakis et al., 2019). Alzheimer’s disease (AD), a major component of age-related dementia, results from progressive neurodegeneration (Lane et al., 2018) characterized by β-amyloid (Aβ) plaques and intracellular neurofibrillary tangles composed of hyperphosphorylated tau protein (Lane et al., 2018).

AD is thought to result from the interplay of genetic susceptibility and unknown environmental factors (Bird et al., 1989; Devi and Scheltens, 2018; Dunn et al., 2019). The insidious and progressive nature of AD makes it difficult to diagnose (Swallow, 2017), and confirmation of AD is based on post-mortem evaluation of cerebral tissues (Weller and Budson, 2018). The lack of effective therapeutic options for AD has shifted research focus toward preclinical AD prediction (Imtiaz et al., 2014; Swallow, 2017). Although evaluation of biomarkers in cerebrospinal fluid was shown to be reliable for predicting AD (Paterson et al., 2018), it requires lumbar puncture and thus is highly invasive (Khan and Alkon, 2015). Therefore, it is imperative to find an alternative diagnostic approach that not only causes less harm to patients but also provides early warning signals of impending AD. Mild cognitive impairment (MCI) is considered an intermediate stage between normal aging and AD (Geda, 2012) that confers a 10–15% annual risk of converting to probable AD (Risacher et al., 2009).

Peripheral biomarker (blood) testing offers advantages over traditional AD screening procedures in terms of cost and invasiveness (Sabbagh and Blennow, 2019). Previous studies have placed emphasis on evaluating known AD biomarkers, such as Aβ or tau isoforms in various peripheral body fluids, such as saliva (Sabbagh et al., 2018) or serum (Li and Mielke, 2019). A longitudinal blood transcriptomic study identified a consistent downregulation of *TOMM40* (translocase of outer mitochondrial membrane 40 homolog) in AD patients and upregulation of several leukocyte-specific genes among those with rapidly vs. slowly advancing disease, including *KIR2DL5A* (killer cell immunoglobulin-like receptor, two domains, long cytoplasmic tail, 5A), *SLC2A8* (solute carrier family 2, facilitated glucose transporter, member 2), and *PLOD1* (procollagen-lysine 2 o-oxoglutarate 5-dioxygenase 1; Chong et al., 2013).

Since mRNA alterations have an unknown impact at the protein level, we designed a proteomic study that sought novel exosome-associated proteins that might serve as peripheral blood biomarkers for MCI/AD progression. Exosomes are a group of endocytosis-related membrane vesicles that act as intercellular messengers by carrying various cargo biomolecules from donor cells to recipient cells (Sancho-Albero et al., 2020). Due to their pivotal regulatory roles, these nanometer-sized particles are ubiquitously distributed throughout the body and can be detected in a variety of easily accessible biospecimens, including blood, urine, or saliva (Yang et al., 2020). Moreover, given their stability, exosomal biomarkers match or outperform their counterparts in conventional specimens, such as serum or urine, in terms of specificity and sensitivity (Lin et al., 2015). Since exosomes can be released by neurons (Sharma et al., 2019), the diagnostic potential of neuron-derived exosomal biomarkers has drawn interest. Notably, exosomes have been proposed to promote the propagation of AD-associated substances across the brain (Bellingham et al., 2012). Several candidates for AD diagnosis were recently unearthed, such as synapse proteins (including synaptophysin, synaptopodin, and synaptotagmins), down-regulation of which might signal neuronal dysfunction (Goetzl et al., 2016), as well as NPTX2 (neuronal pentraxin 2), which maintains neuronal homeostasis (Goetzl et al., 2018; Watson et al., 2019).

Endeavor has been made to identify blood-derived prognostic markers that change as the AD progresses in its early stage. In a previous study, Chong et al. found a lack of *TOMM40* (translocase of outer mitochondrial membrane 40 homolog) at the transcriptomic level in fast- or slow-AD progressors (AD patients with changes in Clinical Dementia Rating-Sum of Boxes score of ≥2 points or <2 points), along with several leukocytes-specific genes, including *KIR2DL5A* (killer cell immunoglobulin-like receptor, two domains, long cytoplasmic tail, 5A), *SLC2A8* (solute carrier family 2, facilitated glucose transporter, member 2), and *PLOD1* (procollagen-lysine 2 o-oxoglutarate 5-dioxygenase 1) that were specifically elevated in fast-AD progressors (Chong et al., 2013). However, such alterations at the mRNA level are not representative of the biological impact at the protein level; in addition, the mere focus on biomarkers of early-stage AD does not reflect the progressive nature of AD. Therefore, the present study employs quantitative proteomic and bioinformatic tools to compare and contrast neuron-derived exosomes in peripheral blood collected from Chinese patients undergoing normal aging and from individuals with diagnoses of MCI and AD.

## Materials and Methods

### Reagents

The ExoQuick ULTRA exosome isolation kit was purchased from SBI System Bioscience (Palo Alto, CA, USA). Anti-L1CAM biotinylated antibody was purchased from R&D Systems (Minneapolis, MN, USA). The human CD81 (Cluster of Differentiation 81) antigen ELISA kit was purchased from CUSABIO (Wuhan, China). ELISA kits for C7 (Complement component 7), FERMT3 (Fermitin Family Member 3), CAP1 (Adenylyl cyclase-associated protein 1), ENO1 (Enolase 1), and ZYX (Zyxin) were purchased from CLOUD-CLONE (Wuhan, China). Albumin/IgG removal kits were purchased from Merk (Shanghai, China). TMT 6-plex labeling kit, MicroBCA protein quantification kit, Streptavidin Plus UltraLink™ Resin, M-PER Mammalian Protein Extraction Reagent, 1M TEAB, and 50% Hydroxylamine were purchased from Thermo Scientific (Rockford, IL, USA). Sequence grade trypsin was purchased from Promega (Madison, WI, USA). Dithiothreitol and indole-3-acetic acid were purchased from GE Healthcare (Shanghai, China).

### Subject Selection and Serum Collection

The overall study design is shown in Supplementary Figure 1. The study was reviewed and approved by the Ethics Committee of Shenzhen Center for Disease Control and Prevention, and all participating subjects provided written informed consent. Subjects were selected from the elderly (60+ years) population in a hospital in Shenzhen. Cognitive status was measured by Mini-cog and MMSE (Mini-Mental State Exam) assessment.

Subjects with Mini-cog score less than 5 and MMSE score ≤21 (for subjects with an education level of primary school or below) or MMSE score ≤24 (for subjects with an education level of secondary school and above) were considered to have MCI (Katzman et al., 1988). Patients were diagnosed with AD by experienced neurologists in accordance with criteria adopted by the U.S. National Institute of Neurological and Communicative Disorders and Stroke Alzheimer’s Disease and Related Disorders Association (NINCDS-ADRDA). A total of five subjects with AD, five with MCI, and five normal age-matched controls participated in this study. Venous blood samples (5 ml) were collected from each subject, and the serum was separated by centrifugation (3,000 rpm for 10 min at 4°C).

### Isolation of Neuron-Derived Exosomes

Total exosomes were separated from 250 μl serum samples and cleaned up by ExoQuick ULTRA kit following the manufacturer’s instruction. One microgram anti-L1CAM biotinylated antibody was linked to 20 μl streptavidin resin by incubation at room temperature for 1 h. Antibody-linked resins were washed in Hank’s balanced salt solution (HBSS), incubated overnight at 4°C with 350 μl cleaned serum exosome solution (pH 8.0), and washed again with HBSS buffer. Finally, the neuron-derived exosomes were eluted with Tris-HCl (pH 3.0).

### Identification of Neuron-Derived Exosomes

Exosome suspension (20 μl) was dropped onto a 400-mesh copper grid and air-dried for 30 min, following which the samples were stained with 20 μl 1% uranium acetate solution for 1 min. The remaining liquid was removed from the copper grid, which was then loaded into a JEM F200 (JEOL, Tokyo, Japan) transmission electron microscope. Ultrastructural images were captured with a resolution of ~100 nm. NTA (Nanoparticle Tracking Analysis) measurements were performed by injecting the samples into a NanoSight LM20 (NanoSight, Amesbury, UK) equipped with a 640-nm laser, and a Viton fluoroelastomer O-ring. Particle size was evaluated with NTA 2.3 software.

### Proteomic Analysis

Neuron-derived exosomal proteins were extracted with Mammalian Protein Extraction Reagent (M-PER). Total protein levels were quantified using a bicinchoninic quantification assay kit; the protein suspension was subsequently filtered by 3 kDa filtration devices (Millipore, CA, USA). Protein reduction and alkylation employed 400 μl DTT solution (100 mM TEAB with 10 mM DTT) and incubation with 400 μl IAA solution (100 mM TEAB with 20 mM IAA), respectively. Next, the proteins were digested with trypsin at 37°C for 15 h; the digestion products were labeled with TMT (6-plex) labeling reagent and samples from different groups were pooled. Peptides were separated by a Dionex™ nano liquid chromatography system (3 μm, 100 Å, 75 μm i.d. ×15 cm, Acclaim PepMap100, C18) and analyzed by high-resolution Orbitrap mass spectrometry (Q-Exactive System, Thermo Scientific, MA, USA). Data were processed and searched against the Uniprot human protein database (a total of 71,434 entries) with Proteome Discoverer 2.1. All groups of reporter ion intensities were log2 transformed to form an expression matrix for bioinformatics analyses. The mass spectrometry proteomics data have been deposited to the ProteomeXchange Consortium (Deutsch et al., 2020) *via* the PRIDE (Perez-Riverol et al., 2019) partner repository with the dataset identifier PXD027561.

### Soft Clustering of AD Progression-Related Proteins

To investigate overall protein expression patterns, a soft clustering method called fuzzy c-means clustering (Kumar and Futschik, 2007) was performed using the R package *Mfuzz* (version 2.48.0). Briefly, the mean protein expression value per stage (normal/MCI/AD) was first computed and fed to the fuzzy c-means algorithm; this resulted in six neuron-derived exosomal protein clusters, each with distinct expression pattern. Protein clusters that showed AD-progression-dependent increase/decrease (defined as bridge clusters) were visualized by heatmap using R package *ComplexHeatmap* (version 2.4.3) and chosen for further analyses. Proteins in bridge clusters were defined as bridge proteins.

### Function Enrichment Analysis

To illuminate the biological impacts caused by altered protein expression across various stages of AD development, the exosomal proteins in 6 clusters were subjected to function enrichment analysis. By searching against the GO/KEGG database (2021, February 11th) using the R package *clusterprofiler* (version 3.16.1), the joint effect of proteins in different clusters was interpreted. The top five enriched GO terms (under Biological process, BP; Molecular function, MF or Cellular component, CC branches) and KEGG pathways with the smallest *q*-value (*p*-value adjusted by Benjamini–Hochberg procedure) were extracted and displayed in bubble plots using R package *ggplot2* (version 3.3.3). The size of the intersection between/among different clusters was displayed in an upset plot using R package *ComplexHeatmap*, where GO terms/KEGG pathways co-regulated by ≥3 clusters (defined as core pathways) were intuitively visualized. Next, the co-regulatory relationship between core pathways and 6 clusters was visualized in a Sankey diagram using the R package *ggalluvial* (version 0.12.3). Finally, a chord diagram showing the co-regulated pathways shared by bridge clusters was generated using R package *circlize* (version 0.4.12).

### Differential Expression Analysis

Differential expression analysis was performed using a *t*-test approach; the DEPs were shown in volcano plots using R package *ggplot2*. The log2-fold-change of a protein between two groups of samples was calculated based on the arithmetic mean of log2-transformed protein expression, which was expressed using the formula:

$$log2FC=\frac{\left(\sum_{i=1}^{m}log2(G1_{i})\right)}{m}-\frac{\left(\sum_{j=1}^{n}log2(G2_{j})\right)}{n}$$

where *G*1_i_ and *G*2_j_ represent the expression of the protein in the *i*th sample of group 1 and the *j*th sample of group 2, respectively. m and n correspond to the total number of samples within group 1 and group 2, respectively. This is equivalent to the log2-transformed ratio of geometric means of the protein expression (original scale) between two groups:

$$log2FC=log2\left(\frac{{\left(\Pi_{i=1}^{m}G1_{i}\right)}^{\frac{1}{m}}}{{\left(\Pi_{j=1}^{n}G2_{j}\right)}^{\frac{1}{n}}}\right).$$

Proteins that satisfied *p*-values <0.05 and log2-fold-change greater than or equal to 0.5 were considered DEPs. The intersection between DEPs and bridge proteins was defined as key proteins and used in experimental validation by ELISA.

### ELISA Assay

After extraction of neuron-derived exosomal protein, the CD81 levels in peripheral blood samples were assayed using the Human CD81 antigen ELISA kit. The best-fit curve of optical density vs. concentration was drawn using Curve Expert software (version 1.3), enabling the calculation of the peripheral blood CD81 concentration. After the bioinformatics analyses, the expression levels of key proteins (C7, FERMT3, CAP1, ENO1, and ZYX) were validated in an independent set of samples using corresponding ELISA kits. The relative protein expression of key proteins (normalized to the levels of CD81) was shown in box plots using the R package *ggpubr* (version 0.4.0), and the *p*-value and statistical significance were displayed by the R package *rstatix* (version 0.6.0).

### Protein-Protein Interaction (PPI) Network Analysis

The PPI network of bridge proteins was established by STRING online database (string-db.org, version 11.0) and analyzed locally with a Cytoscape (version 3.8.1) plug-in called “NetworkAnalyzer” (version 4.4.6). Two topological parameters, namely, the degree of centrality (how many edges are linked to the node) and the betweenness centrality (how frequent the node serves as a bridge in the shortest path between two other nodes) were used to visualize the PPI network.

## Results

### Neuron-Derived Exosomes Were Successfully Isolated From the Peripheral Blood

**Figure 1:**
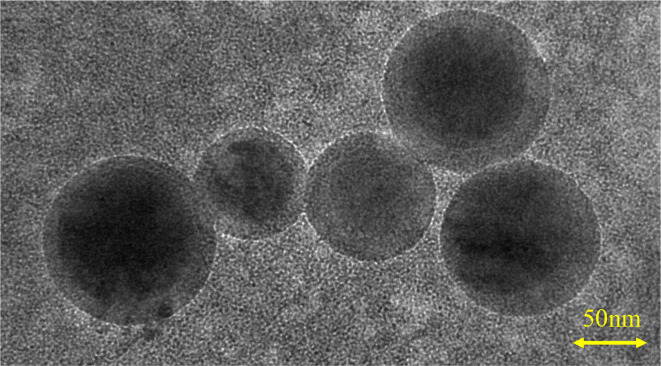
Identification of neuron-derived exosomes. Image showing the double-layer membrane structure of exosomes, with a scale bar of 50 nm.

Transmission electron microscopy revealed ~100 nm exosome particles with double membranes (Figure 1). Nanoparticle tracking analysis suggested that the average size and mean intensity of the particle were 56.19 nm and 66.3%, respectively (Supplementary Figure 2A). The standard curve of CD81 concentration vs. optical density (Supplementary Figure 2B) was later used to calculate the concentration of CD81. As shown in Table 1, a certain concentration of CD81 was detected in peripheral blood samples, indicating the successful isolation of exosomes.

**Table 1:** Evaluation of exosome specific marker CD81 in the peripheral blood sample.

	S0	S1	S2	S3	S4	S5	S6	Sample
OD	0.214	0.235	0.293	0.367	0.439	0.517	1.524	0.337
ng/ml	0.000	0.156	0.312	0.625	1.250	2.500	5.000	0.465

*S0-S6: Serially diluted standard samples*.

### Two Protein Expression Patterns Are Associated With AD Progression

**Figure 2:**
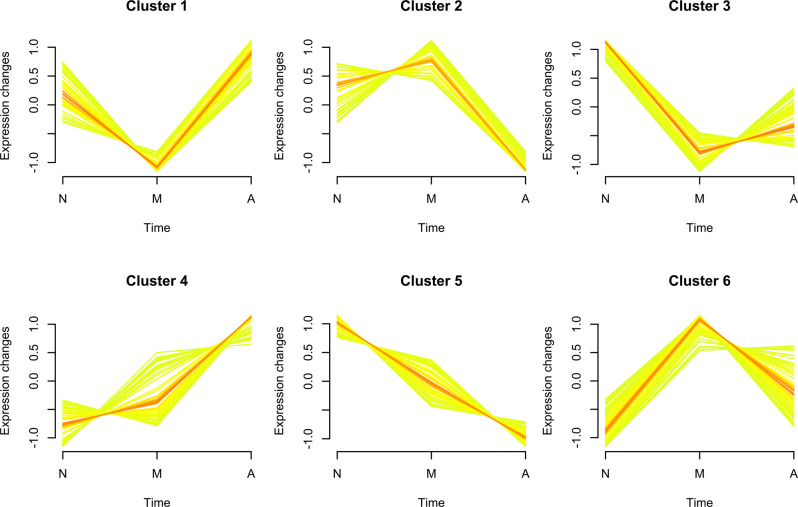
Soft clustering of exosomal protein expression patterns. The membership values of each cluster protein are color-encoded in the plots. Yellow- or green-colored trend lines correspond to bridge proteins with relatively low membership value, whereas their red-colored counterparts represent bridge proteins with higher value of membership. Three characters “N,” “M,” and “A” on the horizontal axis represent normal, mild cognitive impairment (MCI) and Alzheimer’s disease (AD). The six soft clusters exhibit distinct expression patterns of neuron-derived exosomal proteins across the different stages of AD progression, among which Cluster 4/Cluster 5 display a consistently increasing/decreasing trend.

We identified 360 neuron-derived exosomal proteins by tandem mass tag (TMT) quantitative proteomics. The longitudinal evolution of the mean expression of exosomal proteins along the three steps of cognitive decline was assessed by fuzzy c-means algorithm. As shown in Figure 2, the expression patterns of several protein clusters were represented by colored trendlines, and Cluster 4 and Cluster 5 displayed a trend of gradually increasing/decreasing protein expression over the period of AD progression (these clusters were defined as bridge clusters). The proteins in these clusters were named bridge proteins and subjected to subsequent investigations. A list of identified proteins was provided in Supplementary Table 1. The phenotypic information of the subjects used for proteomic analysis is provided in Supplementary Table 2. All results of fuzzy c-means clustering are provided in Supplementary Table 3.

### Function Enrichment Analyses Revealed Several Core Pathways

**Figure 3:**
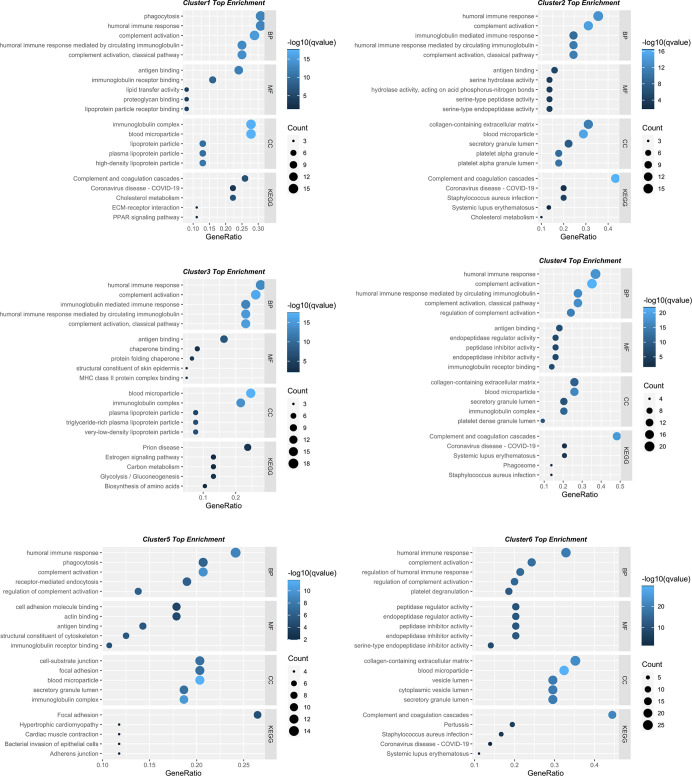
Bubble plots showing the involvement of six protein clusters in different biological processes. The top five enriched KEGG pathways/GO terms for each cluster displayed in bubble plots. The dot size represents the number of proteins involved in each KEGG pathway/GO term, and the color gradient corresponds to the *q*-value-based statistical significance.

**Figure 4:**
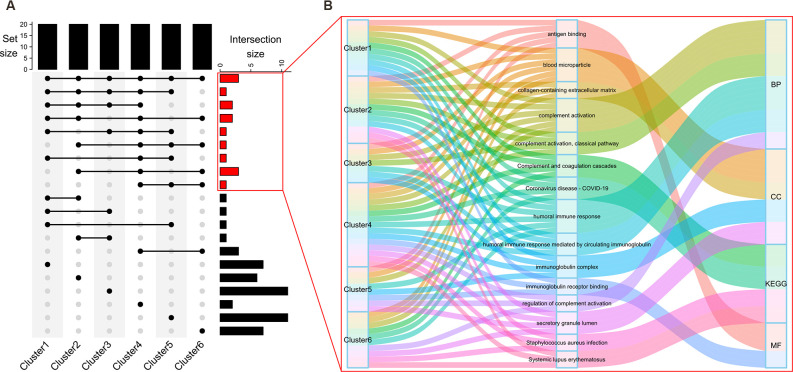
Discovery of core pathways for cognitive decline. The top histogram of the upset plot **(A)** shows the set size of each cluster (five top enriched KEGG pathways plus 5*3 top enriched GO terms under BP, CC, or MF branches, a total of 20 pathways/GO terms). The right histogram represents the size of intersection between two, or among more than two clusters. Specifically, right histograms correspond to the pathways/GO terms co-regulated by the majority (≥3) of clusters were marked in red. Details within these histograms are further depicted in a Sankey diagram **(B)**. Each flow is colored to represent a specific KEGG pathway/GO term. The information flow starts from blocks representing different clusters (source node), passes through the second column of blocks representing the biological processes, and finally converges on one of the four (BP, CC, MF, KEGG) ontologies (sink node).

To gain insight into their biological functions, proteins in all six clusters were used as queries to search GO/KEGG databases. The top enriched pathways/GO terms in each cluster are shown in bubble plots (Figure 3) and further integrated into an upset plot (Figure 4A), wherein the right histograms marked in red correspond to pathways/GO terms co-regulated by ≥3 clusters (core pathways). As shown in Figure 4B, the co-regulatory relationship between core pathways and the 6 clusters were visualized in a Sankey diagram, the most representative core pathways (co-regulated by ≥5 clusters, defined as pivotal pathways) and the corresponding ontologies were “blood microparticle” (GO-CC), “antigen binding” (GO-MF), “complement activation” (GO-BP), and “humoral immune response” (GO-BP).

### Two Bridge Protein Clusters Jointly Regulate Several Biological Pathways

**Figure 5:**
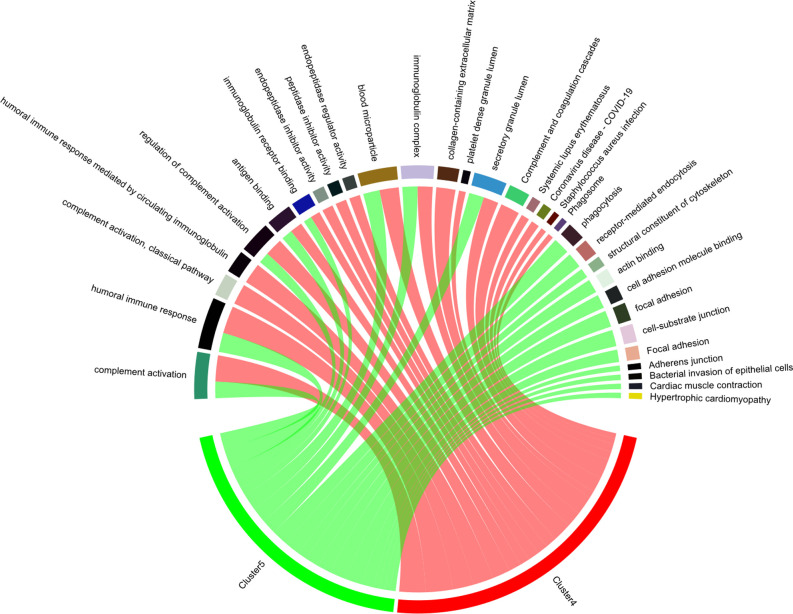
Enriched biological processes of bridge proteins. The participation of bridge proteins in the top five enriched GO terms/KEGG pathways are shown in a chord diagram. The sectors correspond to the top five enriched GO-BP/GO-CC/GO-MF terms and KEGG pathways with the lowest *p*-value. The spill-over from “Cluster 4” or “Cluster 5” sector to other sectors indicates the number of bridge proteins involved in different GO terms/KEGG pathways. Notably, the intersection between pathways regulated by Cluster 4/Cluster 5 bridge proteins are: “complement activation,” “humoral immune response,” “regulation of complement activation,” “antigen binding,” “immunoglobulin receptor binding,” “blood microparticle,” and “secretory granule lumen.”

To investigate the interaction of Cluster 4 and Cluster 5, a chord diagram was used to visualize the involvement of bridge proteins in the top enriched GO terms/KEGG pathways (Figure 5), Cluster 4/Cluster 5 bridge proteins jointly regulate the following biological processes: “complement activation,” “humoral immune response,” “regulation of complement activation,” “antigen binding,” “immunoglobulin receptor binding,” “blood microparticle,” and “secretory granule lumen.” In addition, various pathways are exclusively regulated by Cluster 4 (such as “phagocytosis” and “receptor-mediated endocytosis”) and Cluster 5 (such as “complement activation, classical pathway,” and “humoral immune response mediated by circulating immunoglobulin”).

### K-Means Clustering Based on Expression Profiles of Bridge Proteins Distinguishes the Majority of AD Patients From Their Normal Aging Counterparts

**Figure 6:**
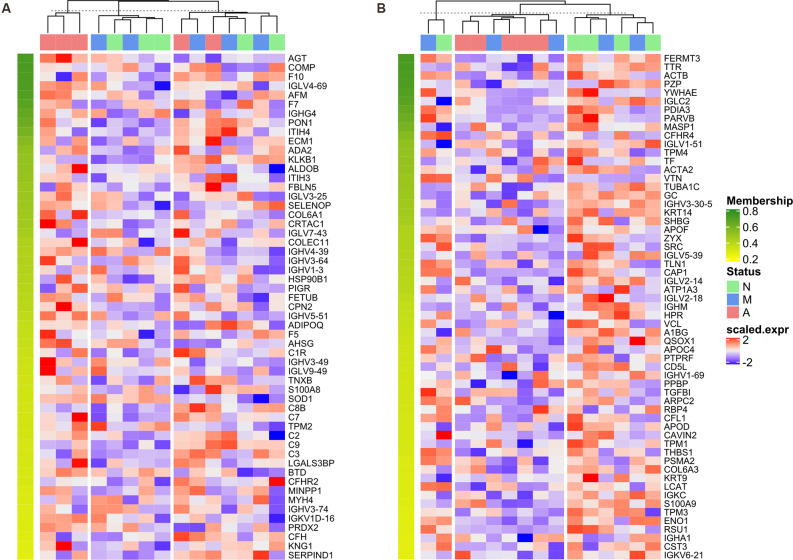
Heatmap of normalized expression matrix of two clusters. The expression matrixes of bridge proteins in Cluster 4 **(A)** and Cluster 5 **(B)** visualized as a heatmap. The dendrogram at the top of the heatmap was generated by K-means clustering based on the expression profile of 55 Cluster 4 bridge proteins/59 Cluster 5 bridge proteins, whereby samples were divided into 3 clusters. The bridge proteins were sorted in descending order according to the corresponding cluster membership; therefore, the membership value decreases from top to bottom of the row annotation (as represented by a color gradient from green to yellow).

There were 55/59 bridge proteins in Cluster 4/Cluster 5, expression of which increased/decreased as cognitive function declined (i.e., from normal to MCI to AD), as shown in the corresponding expression heatmaps (Figures 6A,B). The top dendrogram shows that the K-means clustering was performed based on the expression profile of 55 Cluster 4 bridge proteins/59 Cluster 5 bridge proteins, whereby samples were split into three clusters that distinguish the majority of AD patients from normal controls.

### Several Differentially Expressed Proteins Were Identified by *t*-test

**Figure 7:**
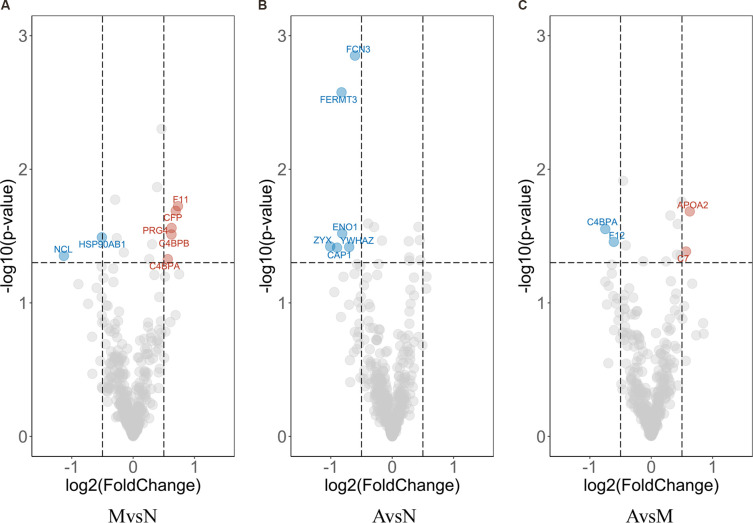
Volcano plot of differential expression analysis. Each dot in the Volcano plot represents a neuron-derived exosomal protein. Difference in expression between two groups and *p*-values are reported as log2 fold changes and –log10 values, respectively. The cut-off limits are set at *p* < 0.05 and log2 fold change ≥0.5, with red/blue dots signifying the up-/down-regulated DEPs calculated by *t*-test of MCI vs. normal group **(A)**; AD vs. MCI group **(B)** and AD vs. normal group **(C)**, respectively. The representative proteins are labeled in the plots.

**Figure 8:**
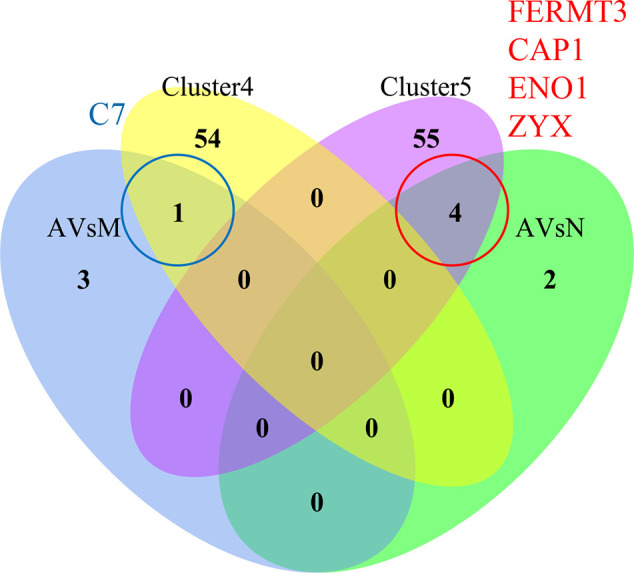
Identification of key proteins in AD progression. The Venn diagram of Cluster 4 (yellow)/Cluster 5 (purple) bridge proteins and DEPs obtained through differential expression analysis between each pair of groups: AD vs. MCI (AVsM, blue)/AD vs. normal (AVsN, green). There is no intersection between Cluster 4/Cluster 5 and DEPs obtained through differential expression analysis of MCI vs. normal. The intersection between Cluster 4 and AVsM was C7, while the intersection between Cluster 5 and AvsN was FERMT3, CAP1, ENO1, and ZYX.

Next, we used a *t*-test approach to determine neuron-derived exosomal proteins with statistically significant changes between each possible combination of the groups. As shown in Figure 7, volcano plots demonstrated the results of pairwise comparison between MCI and normal, AD and normal, and AD and MCI. The DEPs between MCI and N were NCL (Nucleolin), HSP90AB1 (Heat Shock Protein 90 Alpha Family Class B Member 1), F11 (coagulation factor XI), CFP (Cyan Fluorescent Protein), PRG4 (Proteoglycan 4), C4BPB (Complement Component 4 Binding Protein Beta) and C4BPA (Complement Component 4 Binding Protein Alpha; Figure 7A); the DEPs between AD and normal were FCN3 (Ficolin 3), FERMT3, ENO1, ZYX, YWHAZ (Tyrosine 3-monooxygenase/tryptophan 5-monooxygenase activation protein zeta), and CAP1 (Figure 7B); the DEPs between AD and MCI were C4BPA (Complement Component 4 Binding Protein Alpha), F12 (Coagulation Factor XII), APOA2 (apolipoprotein A-II), and C7 (Figure 7C). To improve the robustness of the proteomic results, intersections between bridge proteins in Cluster 4/Cluster 5 and DEPs between two groups of samples were defined as key proteins (Figure 8), including C7 (in Cluster 4, showing a consistent upward trend across the progressive steps of cognitive decline), FERMT3, CAP1, ENO1, and ZYX (in Cluster 5, showing a consistent downward trend across the progressive steps of cognitive decline). Results of *t*-tests were provided in Supplementary Table 4.

### Expression Patterns of C7 and ZYX Were Validated by ELISA Analyses

**Figure 9:**
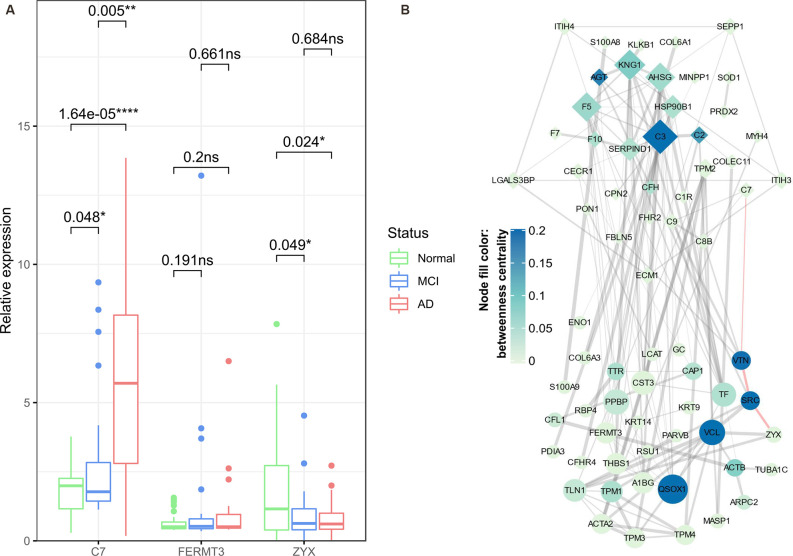
ELISA validation of key proteins and construction of protein-protein interaction (PPI) network. Box and Whisker plot **(A)** demonstrate the median expression (middle line), minimum/maximum expression (whiskers) of key proteins (C7, FERMT3, and ZYX) validated in an independent set of samples using ELISA assay; the outliers are shown as colored dots. The statistical significance of *t*-tests between any two groups of samples is indicated by *p*-values and asterisks (*p < 0.05, **p < 0.01, ****p < 0.0001). The PPI network **(B)** is constructed based on Cluster 4/Cluster 5 bridge proteins (denoted by diamond/oval-shaped nodes). The node size is proportional to the degree centrality of a node, whereas the color gradient represents the betweenness centrality of a node. The width of edges corresponds to the strength of the evidence supporting the connection between two nodes; the red-highlighted edges indicate a potential C7-VTN-SRC-ZYX cascade in the progression of AD. ns, non-significant.

The expression of key proteins was validated in an independent set of samples (containing 32 AD patients, 34 MCI sufferers, and 52 normal aging controls) using ELISA assay, among which the expression of CAP1 and ENO1 was below the detection limits. Hence, only expression of C7, FERMT3, and ZYX was used to generate box and whisker plots (Figure 9A) Statistically significant expression changes of C7 and ZYX were found between different group pairs (except for ZYX expression between MCI and AD), which was almost consistent with the proteomic analysis results. The phenotypic information of the subjects in the independent cohort is provided in Supplementary Table 5.

### PPI Network of Bridge Proteins Reveals a Potential Regulatory Axis

Finally, in the PPI network obtained from the STRING database (Figure 9B), we found that C7 and ZYX might be connected by a path containing VTN (Vitronectin) and SRC (Proto-oncogene tyrosine-protein kinase Src). Although VTN and SRC were not identified as differential enrichment proteins, based on their relatively high betweenness centrality, we speculated that VTN and SRC might exert important anti-AD roles (as they belong to Cluster 5) by regulating several bridge proteins, and most importantly, they might constitute a regulatory axis together with the preceding two key proteins (C7 and ZYX).

## Discussion

We used a TMT-based quantitative proteomic approach to identify and compare neuron-derived blood exosomal markers in participants with AD, MCI, and age-matched healthy controls with the goal of identifying a stepwise progression from normality to AD. First, several expression behaviors of neuron-derived exosomal proteins were identified by fuzzy c-means clustering, whereas the trend lines of Cluster 1 and Cluster 3 exhibited a concaved shape, with minimum expression changes at the MCI stage, in distinct contrast to the expression patterns of bridge proteins in Cluster 2 and Cluster 6 (demonstrated by convex-shaped trend lines). The aforementioned clusters might exert a dual role in the progression of AD (*vide infra*, Liu et al., 2014). By comparison, more consistent trends of elevation/reduction of protein expression were observed in Cluster 4 and Cluster 5.

Despite the divergence among these clusters, we used function enrichment analyses to describe their respective roles in the pathogenesis of AD. The results were visualized using bubble plots, among which the core pathways (GO terms/KEGG pathways co-regulated by ≥3 clusters) were identified and marked red in an upset plot; the corresponding detailed information was further visualized by a Sankey diagram, the most representative core pathways (GO terms/KEGG pathways co-regulated by ≥5 clusters, defined as pivotal pathways) were “blood microparticle,” “antigen binding,” “complement activation,” and “humoral immune response.” Aside from the enrichment in the “blood microparticle” that might be attributed to the source of the samples analyzed (peripheral blood), enrichment of protein clusters in the three remaining pivotal pathways was consistent with evidence that neuroinflammation contributes to the pathogenesis of AD (Heneka et al., 2015). Antigen binding is a fundamental process for the initiation of various immune effector functions, including phagocytosis and neutralization of receptors (Heesters et al., 2016). Complement activation is a crucial innate immune process for timely recognition and clearance of exogenous pathogens and endogenous misfolded proteins (Ricklin and Lambris, 2013). In the context of AD, the complement system could compensate for the insufficient clearance of Aβ and trigger relevant adaptive immune responses (Tenner, 2020). The B cell-mediated humoral immune response, which was initiated by antigen binding to clonally distributed B-cell receptors (Liu et al., 2010), was thought to bear anti-AD potential: as early as 1993, four AD patient-derived B cell lines were found to secret antibodies that target Aβ peptide in a specific manner (Gaskin et al., 1993), an in-depth animal study demonstrated that the formation of amyloid plaques was abolished by Aβ immunization (Schenk et al., 1999), further corroborating the therapeutic potential of the humoral immune response in Alzheimer disease. Although we found several pivotal pathways that might be co-regulated by different protein clusters, our purpose was to identify proteins accountable for AD-progression; therefore, we focused on Cluster 4 and Cluster 5 that display a consistently increasing/decreasing trend, as these clusters might promote or impede the development of AD. Thus, the combined action of Clusters 4 and 5 was further investigated.

The chord diagram indicated that several enriched GO terms/KEGG pathways were co-regulated by bridge proteins in Cluster 4 and Cluster 5. Both clusters were highly implicated in immune-associated pathways including “complement activation,” “antigen binding,” “immunoglobulin receptor binding,” “immunoglobulin complex,” and “humoral immune response.” Since “antigen binding,” “complement activation,” and “humoral immune response” were previously defined as pivotal pathways, this suggests that clusters other than Clusters 4 and 5 might also participate in these pathways. However, “immunoglobulin receptor binding” and “immunoglobulin complex” might be more specifically regulated by Cluster 4/Cluster 5. Immunoglobulin is important in AD pathology, as illustrated by the significantly lower serum IgG autoantibody level relative to healthy controls (Acharya et al., 2013) although, on the other hand, an inverse correlation was proposed between cerebral Aβ burden and IgM in a mouse model of AD (Wang et al., 2017). Taken together, the findings underscore the role of the immune system (especially immunoglobulin-associated immune processes) in the evolution of AD. In our subsequent heatmap analysis, the expression profiles of bridge proteins in Cluster 4/Cluster 5 were used for K-means clustering, which distinguished most AD subjects from their normal aging counterparts. These results highlight the biological significance of the bridge proteins.

Next, we performed differential protein expression analysis, in addition to time-course analysis, to ensure the statistical rigor of this study. We defined several key proteins based on the intersections between bridge proteins and differential enrichment proteins (DEPs). The biological roles of representative identified DEPs included two major constituents of C4BP (complement component 4 binding proteins), namely, C4BPA and C4BPB, that were differentially expressed between the MCI (elevated) and the normal aging group. However, the elevated expression in MCI samples was not totally consistent with the cerebral-protective roles of C4BP, such as reducing excessive complement activation mediated by extracellular Aβ accumulation (Trouw et al., 2008). Nevertheless, we hypothesized that this phenomenon might be an innate resistance, although perhaps not sufficient in magnitude, to counteract AD progression. Second, we have ENO1, which is a crucial glycolytic enzyme (Butterfield and Lange, 2009) differentially expressed between AD (reduced) and the normal aging group. In a previous report, proteomic results suggested that ENO1 was prone to oxidation in the cerebral tissue of both 3×Tg-AD mice and AD patients (Shen et al., 2015); such modification might result in the altered metabolic processing of glucose and degradation of Aβ (Butterfield and Lange, 2009). Finally, APOA2 was differentially expressed between the AD (elevated) and MCI group, and is the second major apolipoprotein of the high-density lipoprotein cholesterol (HDL-C). This protein inhibits cholesterol efflux by regulating the activity of several enzymes associated with HDL-C remodeling (Bandarian et al., 2016). Importantly, a reduced plasma APOA2 protein level was previously found to be related to cognitive decline in normal aging subjects during a 2-year follow-up period (Song et al., 2012); this suggests that the relatively low APOA2 expression in the MCI group were predisposed to further decline in their cognitive function.

Among five key proteins, ELISA validation of C7 and ZYX was almost consistent with the proteomic results. By utilizing whole-exome sequencing (WES) technology, *C7* was previously identified as a risk gene for AD in the Han Chinese population (Zhang et al., 2019): first, an exome-wide missense variant rs3792646 was identified in the *C7* gene, the corresponding risk allele rs3792646-C might exert potential influence over the working memory performance, as well as the cerebral structure of the carriers (e.g., reduced volume of the right hippocampus); second, at the transcriptomic level, *C7* was the only elevated component of the terminal complement complex in brain tissues of AD patients; such transcriptional change was concordant with the ELISA-validated expression pattern of C7 in this study, and we hypothesized that the neuron-derived exosomes might be responsible for the dissemination of C7 protein from the brain to the peripheral vascular system. ZYX is predominantly expressed during brain development (Fujita et al., 2009); its subcellular localization is decisive for whether it promotes (Hervy et al., 2010) or prevents (Kato et al., 2005) apoptosis. ZYX contributes to the stability of HIPK2 (homeodomain interacting protein kinase 2); HIPK2 promotes apoptosis in the DNA damage response process by forming a complex with p53 and inducing phosphorylation at serine 46, thereby triggering the expression of multiple pro-apoptotic regulators (Crone et al., 2011). In the context of AD, Lanni et al. (2013) found that two Aβ peptides could suppress the expression of ZYX, thereby inhibiting the activity of HIPK2, and indirectly modulating apoptosis by inducing the unfolded conformation of p53 that impedes the normal apoptotic process in the presence of stimulation. Such observation (Aβ induced ZYX degradation) might explain the gradually decreasing expression of neuron-derived ZYX over the period of AD progression in this study.

Although significant results were found, it should be noted that there are some limitations to this study. First, we did not perform phosphoproteomics, therefore, phosphorylation of tau (e.g., pT181 and pS396) was not identified. Second, this study was designed to explore novel AD markers, which led to the neglect of known AD markers (such as the level of Aβ). The lack of evidence on tau phosphorylation and Aβ level makes it difficult to associate the current findings with the empirical knowledge about AD, which may potentially impact the interpretation of our current results. Third, APOA2 was found to be a DEP in this study, which might signal co-isolation of lipoproteins. In addition, only one extracellular vesicles marker (membrane protein CD81) was used to determine the successful isolation of the exosomes. Finally, given the relatively small sample size for proteomic analysis (5 normal aging controls, 5 MCI sufferers, and 5 AD patients) and ELISA assay (52 normal aging controls, 34 MCI, and 32 AD patients), validation of the present findings is needed by longitudinal studies of a larger number of subjects progressing from MCI to AD.

In summary, transcriptomic expression of the *C7* gene was previously identified as a risk factor for AD (Song et al., 2012), whereas ZYX was known to be degraded by Aβ peptides in the neuroblastoma cell model (Lanni et al., 2013). Hence, in the current study, for the first time, we demonstrate that C7 (at protein level) and ZYX (in human sample) might be novel neuron-derived protein markers for cognitive decline.

## Data Availability Statement

The datasets presented in this study can be found in online repositories. The mass spectrometry proteomics data have been deposited to the ProteomeXchange Consortium via the PRIDE partner repository with the dataset identifier PXD027561.

## Ethics Statement

The studies involving human participants were reviewed and approved by the ethics committee of Shenzhen center for disease control and prevention. The patients/participants provided their written informed consent to participate in this study.

## Author Contributions

JL and FZ proposed the topic, and conceived and designed the study. WL, SW, YL, LN, RL, XT, and XY contributed to the data collection. XR and SW performed the experiments and contributed to proteomic data processing. JZ and XR drafted the manuscript and contributed to the down-stream bioinformatic analyses and data visualization. All authors contributed to the article and approved the submitted version.

## Conflict of Interest

The authors declare that the research was conducted in the absence of any commercial or financial relationships that could be construed as a potential conflict of interest.

## Publisher’s Note

All claims expressed in this article are solely those of the authors and do not necessarily represent those of their affiliated organizations, or those of the publisher, the editors and the reviewers. Any product that may be evaluated in this article, or claim that may be made by its manufacturer, is not guaranteed or endorsed by the publisher.

## Supplementary Material

SUPPLEMENTARY FIGURE 1 Schematic diagram of the current study.

SUPPLEMENTARY FIGURE 2 **(A)** Results of nanoparticle tracking analysis. **(B)** The standard curve of CD81 concentration vs. optical density.

SUPPLEMENTARY TABLE 1 The list of identified proteins.

SUPPLEMENTARY TABLE 2 Phenotypic information of all subjects used in proteomic analysis.

SUPPLEMENTARY TABLE 3 The results of fuzzy c-means clustering, including the assignments of exosomal proteins and the membership of exosomal proteins in different clusters. The standard deviation of each exosomal protein expression within a certain group (Normal, MCI, or AD) is also shown.

SUPPLEMENTARY TABLE 4 Detailed results of the DEPs between different stages of cognitive decline.

SUPPLEMENTARY TABLE 5 Phenotypic information of all subjects in ELISA validation cohort.
