# Supplementary material for: Discovery of Novel Markers for Identifying Cognitive Decline Using Neuron-Derived Exosomes
Source: Front Aging Neurosci. 2021 Aug 26;13:696944. doi: 10.3389/fnagi.2021.696944 (PMC8427802; doi:10.3389/fnagi.2021.696944)

Supplementary Figure S1

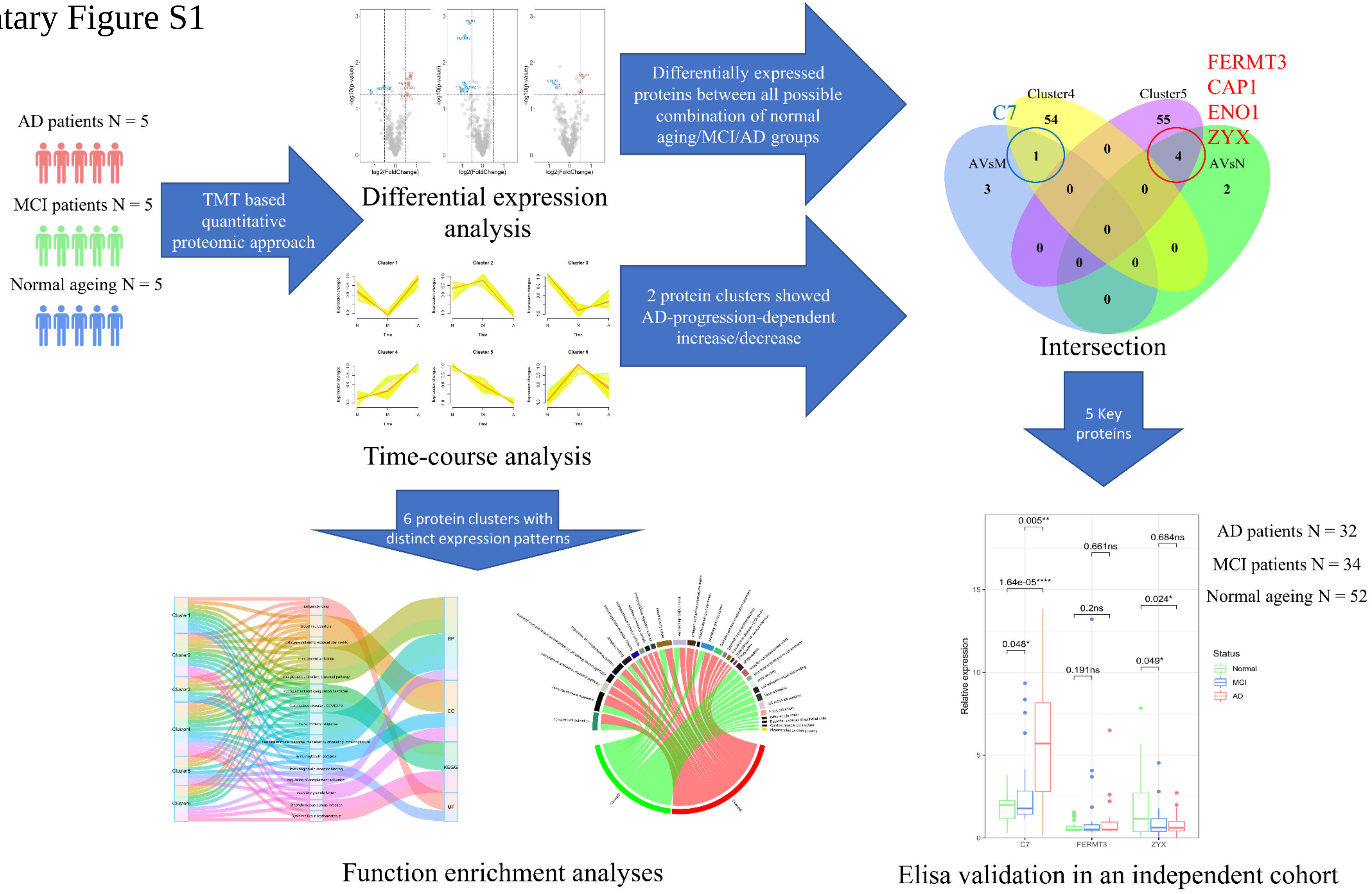

Supplementary Figure S2

A

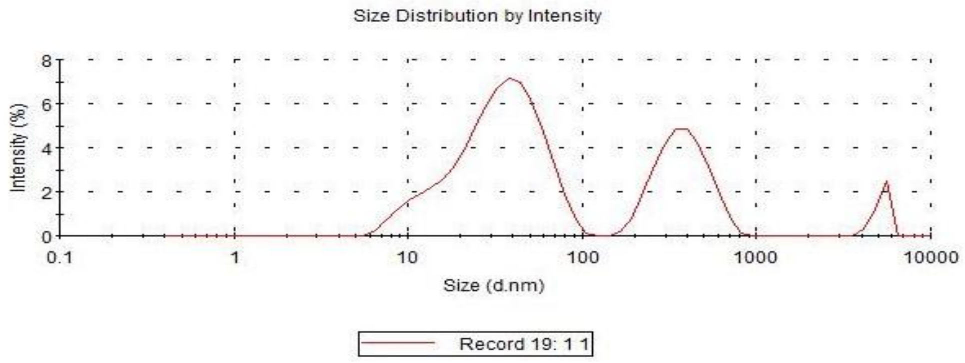

			Size (d.nm):	% Intensity	Width (d.nm):
Z-Average (d.nm):	56.19	Peak 1:	36.31	66.3	18.66
PdI:	0.495	Peak 2:	388.4	29.7	126.1
Intercept:	0.922	Peak 3:	5227	4.0	458.6
Result quality :	Refer to quality report				

B

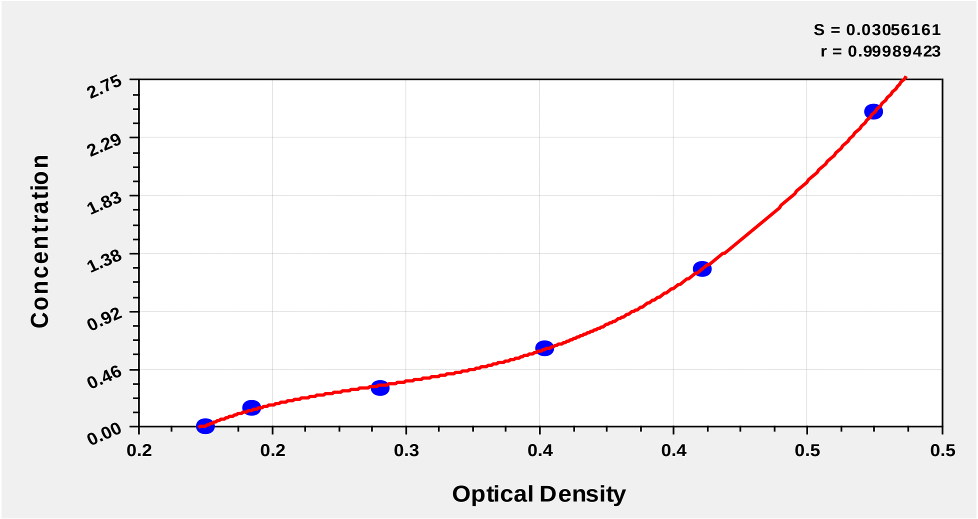

Supplement: SUPPLEMENTARY FIGURE 1 — Schematic diagram of the current study. [file Data_Sheet_1.pdf]
